# Identification of Gut Microbiota Profile Associated with Colorectal Cancer in Saudi Population

**DOI:** 10.3390/cancers15205019

**Published:** 2023-10-17

**Authors:** Areej A. Alhhazmi, Yahya A. Almutawif, Walaa A. Mumena, Shaima M. Alhazmi, Turki S. Abujamel, Ruba M. Alhusayni, Raghad Aloufi, Razan R. Al-Hejaili, Rahaf Alhujaily, Lama M. Alrehaili, Ruya A. Alsaedy, Rahaf H. Khoja, Wassal Ahmed, Mohamed F. Abdelmohsen, Waleed Mohammed-Saeid

**Affiliations:** 1 Medical Laboratories Technology Department, College of Applied Medical Sciences, Taibah University, Al-Madinah Al-Munawarah 42353, Saudi Arabia; ymutawif@taibahu.edu.sa (Y.A.A.); raghad.jaza@gmail.com (R.A.); rahafalhujaily@gmail.com (R.A.); 2 Clinical Nutrition Department, College of Applied Medical Sciences, Taibah University, Al-Madinah Al-Munawarah 42353, Saudi Arabia; wmumena@taibahu.edu.sa; 3 Botany and Microbiology Department, Science College, King Saud University, Riyadh 12372, Saudi Arabia; sh.alhazmi@gmail.com; 4 Vaccines and Immunotherapy Unit, King Fahd Medical Research Center, King Abdulaziz University, Jeddah 21589, Saudi Arabia; tabujamel@kau.edu.sa; 5 Department of Medical Laboratory Sciences, Faculty of Applied Medical Sciences, King Abdulaziz University, Jeddah 21589, Saudi Arabia; 6 Department of Pharmaceutics and Pharmaceutical Technology, College of Pharmacy, Taibah University, Al-Madinah Al-Munawarah 42353, Saudi Arabia; ruba.alhusayni@gmail.com (R.M.A.); razan_alhujili@outlook.sa (R.R.A.-H.); lama.malrehaili@gmail.com (L.M.A.); ruya.alsaedy@gmail.com (R.A.A.); ph.rahafkhoja@gmail.com (R.H.K.); wesalkhaled1420@gmail.com (W.A.); wneyaz@taibahu.edu.sa (W.M.-S.); 7 Department of Clinical Oncology, Faculty of Medicine, Suez Canal University, Ismailia 41522, Egypt; drmohammedfouad80@yahoo.com; 8 Oncology Department, King Fahd Hospital, Ministry of Health, Al-Madinah Al-Munawarah 32253, Saudi Arabia

**Keywords:** colorectal cancer (CRC), microbiota, 16s rRNA, genome, dysbiosis, bacteria, operational taxonomic unit

## Abstract

**Simple Summary:**

Colorectal cancer (CRC) is the third most common malignancy in the world and is the second most common cancer in Saudi Arabia. The faecal-associated microbiota has been dynamically linked to CRC worldwide, which, in turn, may offer evidence for procarcinogenic bacterial effectors-associated with CRC pathophysiology. Studies in gut microbiome and CRC are lacking on Saudi population, which is unique in lifestyle, diet, and genetic backgrounds. Data from CRC-associated intestinal micobiome provided a deeper understanding of CRC and serve as baseline for CRC predicators. In this project, 16S rRNA (V3-V4) gene sequencing analyses coupled with patient’s demographic, diet, and clinical data were deployed to identify microbiota associated with late stages of CRC. Understanding CRC pathophysiology in relation to intestinal microbiota allows early screening of CRC and prognostic option, which has the potential to treat patients at early stage and follow up with patient status. This will result in better outcomes and more cost-effective treatments. This comprehensive approach stratified CRC patients resulting in a potential better health care.

**Abstract:**

Colorectal cancer (CRC) is a significant global health concern. Microbial dysbiosis and associated metabolites have been associated with CRC occurrence and progression. This study aims to analyze the gut microbiota composition and the enriched metabolic pathways in patients with late-stage CRC. In this study, a cohort of 25 CRC patients diagnosed at late stage III and IV and 25 healthy participants were enrolled. The fecal bacterial composition was investigated using V3-V4 ribosomal RNA gene sequencing, followed by clustering and linear discriminant analysis (LDA) effect size (LEfSe) analyses. A cluster of ortholog genes’ (COG) functional annotations and the Kyoto Encyclopedia of Genes and Genomes (KEGG) were employed to identify enrichment pathways between the two groups. The findings showed that the fecal microbiota between the two groups varied significantly in alpha and beta diversities. CRC patients’ fecal samples had significantly enriched populations of *Streptococcus salivarius*, *S. parasanguins*, *S. anginosus*, *Lactobacillus mucosae*, *L. gasseri*, *Peptostreptococcus*, *Eubacterium*, *Aerococcus*, Family XIII_AD3001 Group, *Erysipelatoclostridium*, *Escherichia-Shigella*, *Klebsiella*, *Enterobacter*, *Alistipes*, *Ralstonia*, and *Pseudomonas* (Q < 0.05). The enriched pathways identified in the CRC group were amino acid transport, signaling and metabolism, membrane biogenesis, DNA replication and mismatch repair system, and protease activity (Q < 0.05). These results suggested that the imbalance between intestinal bacteria and the elevated level of the predicated functions and pathways may contribute to the development of advanced CRC tumors. Further research is warranted to elucidate the exact role of the gut microbiome in CRC and its potential implications for use in diagnostic, prevention, and treatment strategies.

## 1. Introduction

Cancer is responsible for almost 10 million deaths globally, making it the second most common cause of death [1,2]. Colorectal cancer (CRC) is a major global health concern that ranks third in terms of the most diagnosed types of cancer, accounting for approximately 10% of all cancer cases per year. Additionally, CRC is the third leading cause of cancer-related deaths worldwide, with nearly a million deaths reported in 2020 (9.4%) [1]. In Saudi Arabia, CRC is a significant health concern; according to recent studies, CRC is the most common cancer among males and the second most common cancer among females in the country [2,3]. Additionally, the incidence rate of CRC in Saudi Arabia has been increasing over the past few years [2,3].

The incidence of CRC is influenced by two types of risk factors: modifiable and non-modifiable factors [4]. The modifiable risk factors include obesity, diets rich in red and processed meat, alcohol consumption, smoking, and inflammatory bowel diseases [4]. The second type is non-modifiable risk factors, such as a family history of CRC or polyps in the colon, hereditary factors, and age. These factors also contribute to an increased incidence of CRC, although at a lower rate than modifiable risk factors [4,5,6,7]. A diet rich in red/processed meat, saturated fats, and refined carbohydrates increases CRC risk, while a high-fiber, high-fruit, and high-vegetable diet protects against CRC by increasing beneficial gut bacteria [8]. The utilization of microbiota-based methods, such as fecal microbiota transplantation (FMT), probiotics, and prebiotics consumption, can play a critical role in lowering the incidence of CRC [9,10]. It is important to point out that CRC occurrence can vary based on the presence of one or more of these risk factors. Nevertheless, individuals who exhibit such risk factors are encouraged to seek appropriate diagnostic measures, as they have a higher likelihood of developing the disease.

Recent studies suggest that dysbiosis (changes in microbial diversity and microbial taxa) in the gut microbiota can cause an imbalance in microbial homeostasis and the proliferation of specific microbes, contributing to CRC development through inflammation, gut barrier damage, and harmful metabolite production [8,11,12]. Other human diseases like obesity, diabetes type II, and inflammatory bowel diseases are known to be significantly affected by the gut microbiota. The etiology of intestinal cancers may be substantially correlated with microbial dysbiosis, according to emerging data [13]. Recent metagenomics-based studies have found that the guts of CRC patients contain enriched populations of *Parvimonas micra*, *Solobacterium moorei*, *Fusobacterium nucleatum*, and *Peptostreptococcus stomatis* [13]. Additionally, enterotoxigenic *Bacteroides fragilis* has been found in higher concentrations in CRC patients’ feces and colonic mucosa [14,15]. In a recent study, Tjalsma et al. presented a bacterial driver–passenger model for CRC pathogenesis, demonstrating that CRC could be established by a “driver” bacterium that is ultimately replaced by a “passenger” bacterium throughout carcinogenesis [13]. The dysbiosis of the microbiota may contribute to the production of oncometabolites and tumor suppressor metabolites, which have adverse impacts on the immune system and genotoxicity [16]. Dysbiosis microbiota can generate a multitude of metabolites that play a significant role in the development of CRC. These include secondary bile acid [17], short chain fatty acid, SCFA, notably lactic acid, acetic acid and propionic acid [18,19], colibactin [20] trimethylamine/Trimethylamine-N-Oxide (TAM/TMAO) [21], and various others compounds. However, it is still unclear how the human gut microbiota and these metabolites contribute to the development of CRC. Understanding the roles played by the microbiome and the metabolome in the pathogenesis of CRC is crucial.

In this study, we determine the gut microbiota profile and the enriched metabolic pathways among individuals who have been diagnosed with CRC at a late stage in Saudi Arabia. Additionally, we assess the association of risk factors, particularly diet quality and fat intake, with CRC and their relationship with microbiota dysbiosis in the gut.

## 2. Materials and Methods

### 2.1. Sample Collection

The fecal specimens of CRC patients (average age = 54.32 ± 14.2) were collected from the Oncology Department at King Fahd Hospital in Al-Madinah Al-Munawarah, Saudi Arabia. The healthy control samples (25 Participants) (average age = 47.40 ± 11.72) were collected from volunteers at Taibah University. There is no significant difference in age between the two groups (*t*-test, *p* = 0.066). The exclusion requirements were similar for CRC and healthy participants, being defined as follows: (1) patients who had abdominal surgery or other invasive treatment within up to months prior to sample collection; (2) patients who took antibiotics, corticosteroids, or probiotics up to three months prior to sample collection; (3) patients with a history of cancer or inflammatory bowel diseases; (4) patients on a special diet; (5) patients who had fecal microscopic examination; and (6) patients diagnosed with diabetes, liver, or kidney diseases. Additionally, CRC patients who used evacuants or underwent colonoscopy up to one week before sample collection were excluded from this study (only for CRC patients). Table 1 (Table S1) displays the demographic features of both CRC and healthy participants. The fecal samples were held at −80 °C until DNA extraction.

All the protocols and procedures of this study were approved by the General Directorate of Health Affairs in Al-Madinah Al-Munawarah (IRB-140-2021) and the Scientific Research Ethics Committee at the College of Applied Medical Sciences (2021/97/118/MLT). All participants signed the informed consent form before participating in the experiment.

### 2.2. Assessment of Diet Quality and Fat Intake

Only 15 and 10 of the 50 participants, who were healthy controls and CRC, respectively, provided data concerning their diet quality and fat intake. Five CRC patients died prior to the interview date, and 10 transferred their treatment to different hospitals. The diet quality of participants included in this study was evaluated using a modified version of the short-form food frequency questionnaire (SFFFQ) that was originally developed by Cleghorn et al. [22]. This tool has been used in previous studies conducted in Saudi Arabia [23,24] and other settings [25,26,27]. Data were collected during face-to-face interviews, and the data collector recorded responses on a hard copy of the questionnaire, which was entered later into an Excel sheet to calculate the diet quality score. The SFFFQ consisted of 20 items, defined as follows: “Fruits”; “Fruit juice”; “Salad”; “Cooked vegetables”; “Fried potatoes/chips”; “Beans or legumes”; “Fiber-rich breakfast cereal”; “Whole wheat bread”; “Cheese/Yoghurt”; “Crisps/Savory snacks”; “Sweet biscuits”; “Ice cream/cream”; “Fizzy drinks/Pop”; “Beef or lamb”; “Chicken or turkey”; “Processed meats/meat product”; “Processed chicken/turkey”; “Fried white fish”; “White fish”; “Oily fish”. Food frequencies for items 1–13 were as follows: “Never or Rarely”; “<1 time per week”; “1 time per week”; “2–3 times per week”; “4–6 times per week”; “≥7 times per week”.

The fat intake of participants was evaluated using a modified version of a food frequency questionnaire (FFQ) that was originally developed by [28]. The FFQ was modified by two experts, and food items commonly consumed by Saudis were added. The FFQ consists of 23 items, defined as follows: “Beef/lamb/camel”; “Chicken (not fried)”; “Fried food (fried chicken, fried fish, kubba, sambosa, fried vegetables, and French fries)”; “Egg (boiled, fried, or scrambled)”; “Fish (salmon or sardine)”; “Processed meat (salami, hotdog, or cold cuts)”; “Sausages”; “Cakes/pastries”; “Chocolate”; “Cookies/biscuits”; “Cream”; “Full-fat cheese/cream cheese”; “Full-fat milk”; “Ice-cream”; “Nuts”; “Tahini/Halawa tahinia”; “Mayonnaise”; “Salad dressing”; “Ghee”; “Butter”; “Plant fats (olive oil, canola oil, corn oil)”; “Avocado”; “Chips”. The frequency of consumption provided in the FFQ was as follows: daily (once a day, 2–3 times per day, 4–5 times per day, 6 or more per day); weekly (once per week, 2–4 per week, 5–6 per week); monthly (1–3 per month, less than once per month).

### 2.3. DNA Extraction and PCR Amplification

DNA was extracted from ~250 mg of stool samples using QIAamp PowerFecal Pro DNA kit (Qiagen, Hilden, Germany), following the manufacturer’s instructions. The concentration of extracted DNA was evaluated using a Qubit 4 fluorometer (Invetrogen, Waltham, MA, USA), and the purity was assessed using a NanoDrop spectrophotometer (Thermo Fisher, Waltham, MA, USA).

The bacterial 16S rRNA gene’s V3–V4 hypervariable regions were amplified using the following primers: Forward primer: 5′-CCTAYGGGRBGCASCAG-3′ and Reverse primer: 5′-GGACTACNNGGGTATCTAAT-3′. All PCR protocols used 15 μL of New England Biolabs’ Phusion^®^ High-Fidelity PCR Master Mix, 2 mM of forward and reverse primers, and approximately 10 ng of template DNA. Denaturation at 98 °C for one minute was followed by 30 cycles of annealing at 50 °C for 30 s, elongation at 72 °C for 30 s, and 72 °C for 5 min. Next, 2% Electrophoresis agarose gel was used to evaluate the PCR results. With the use of a Qiagen Gel Extraction Kit (Qiagen, Hilden, Germany), the PCR products were further purified.

### 2.4. Library Construction

Sequencing libraries were generated using TruSeq^®^ DNA PCR-Free Sample Preparation Kit (Illumina, San Diego, CA, USA), following the manufacturer’s recommendations, and indexes were added. Qubit and real-time PCR were used to quantify the library, and a bioanalyzer was employed to detect size distribution. On Illumina platforms, quantified libraries were pooled and sequenced in accordance with the effective library concentration and required amount of data. The raw data were submitted to the Sequence Read Archive (SRA) of the National Centre for Biotechnology Information (NCBI), under the submission SUB13865299.

### 2.5. 16SrRNA Bioinformatics Analysis

Paired-end readings were assigned to samples by removing the barcode and primer sequence based on their distinctive barcodes. FLASH (V1.2.11, available at http://ccb.jhu.edu/software/FLASH/, accessed on 15 June 2023) was used to combine paired-end reads. [29] defined a minimum length overlap of 10 nucleotides and maximum mismatch of 20%. Quality filtering of the raw tags was performed using the fastp software (Version 0.23.1) with Phred quality ≥ Q19 and a required length of 15 to obtain high-quality Clean Tags [30]. The tags were compared to the reference database (Silva database (16S/18S), https://www.arb-silva.de, accessed on 15 June 2023) using Vsearch (Version 2.16.0) to detect chimera sequences, and then the chimera sequences were removed [31]. Then, the effective tags were finally obtained. The Deblur module in the QIIME2 software (Version QIIME2-202006) was used to denoise the previously obtained effective tags to generate the first OTUs (operational taxonomic units). Then, OTUs with an abundance of less than 5 were filtered out [32]. The software QIIME2 was used to carry out the species annotation. Silva Database release 111 [33] served as the annotation database. Multiple sequence alignment was carried out using QIIME2 software to analyze the phylogenetic relationships between each OTU and the differences in the dominant species across various samples (groups).

### 2.6. Microbial Data Analysis

The sample with the lowest sequences (60,178 sequences) was used as the reference sequence number to standardize the absolute abundance of OTUs. Based on the output of the normalized data, assessments of alpha diversity and beta diversity were carried out. Alpha diversity profiling was calculated based on 3 indices in QIIME2, namely Chao1, Shannon, and Simpson. Chao1 was selected to identify community richness: Chao—the Chao1 estimator. Two indices were used to identify community diversity: Shannon (the Shannon index) and Simpson (the Simpson index). Additionally, the permutational multivariate analysis of variance (PERMANOVA) was used to determine the beta-diversity (QIIME2), which was illustrated using a PCoA diagram based on Bray–Curtis analysis measured in an unannotated OTUs composition. The two-dimensional PCoA results were presented using the ade4 package and ggplot2 package in R software (Version 2.15.3).

The adonis and anosim features of the QIIME2 program were used to investigate the significance of the variations in community structure between the groups. Additionally, multi-response permutation procedure (MRPP) analysis was performed to determine whether the difference in microbial community structure among groups was significant using the MRPP function of the vegan package for the R software (Version 4.0.3). The R software (Version 3.5.3) was used to perform a *t*-test analysis to determine the significantly different species at each taxonomic level (Phylum, Class, Order, Family, Genus, Species). STAMP software (Version 2.1.3) was used to illustrate the differences in taxa and functional pathways. Applying the Benjamini and Hochberg approach, the false discovery rate (FDR) was used to convert the P value to the Q-value [34]. The Linear discriminant analysis (LDA) effect size (LEfSe) analysis using LEfSe software (Version 1.0) (LDA score threshold: 4) was used to examine intergroup differences at the phylum, class, order, family, genus, and species levels. To assess the significance of differences in OTUs between the two groups, LEfSe employs the two-tailed nonparametric Kruskal–Wallis test. Further, to analyze the functions of the communities in the samples and identify the different functions of the communities in the two groups CRC vs. healthy controls, the PICRUSt2 software (Version 2.1.2-b) was used for function annotation analysis, including a cluster of ortholog genes’ (COG) functional annotation and the Kyoto Encyclopedia of Genes and Genomes (KEGG).

### 2.7. Statistical Analysis

R software (version 4.1.3) and IBM SPSS Statistics (version 22.0) were used for the statistical analyses. Descriptive data for continuous variables were presented as mean ± standard deviation (SD) and median (interquartile range). The normality of distributions was assessed using the Shapiro–Wilk test, and the majority of the continuous variables were skewed. Categorical variables were compared using the Chi-square test. Two clinical groups (CRC and HC groups) were compared regarding differences in continuous variables (age and alpha diversity indices) using an independent *t*-test. The Mann–Whitney U test was used to compare the median across the groups (CRC vs. healthy). Logistic regression (odds ratio and the 95% confidence interval) was used to evaluate the association between diet quality and fat intake based on the health status of the participants. Simple linear regression analysis was performed to explore the association between diet quality and fat intake (predictors) in relation to the most abundant bacteria in CRC. The significance of all tests performed was assessed at the 95% confidence level.

## 3. Results

### 3.1. Clinical Samples

This study cohort encompassed two groups: 25 patients diagnosed with CRC and 25 healthy controls (HC). Male individuals presented with a major prevalence among CRC cases (60%), a similarly healthy group (84%) (Chi square, *p* > 0.99). CRC patients’ and healthy controls’ age averages were 54.32 (±14.19) and 47.40 (±11.72), respectively. There is no significant difference in age between the two groups (*t*-test, *p* = 0.066). Among the CRC patients, body status distribution was as follows: 8% (2 out of 25) were underweight, 48% (12 out of 25) had a normal body weight, 9% (2 out of 25) were classified as obese, and 36% (9 out of 25) were categorized as pre-obese. In contrast, among the healthy group, the most prevalent category was normal weight with 64% (16 out of 25), followed by 28% (7 out of 25) classified as obese and 8% (2 out of 25) categorized as pre-obese. Two participants (8%) among the CRC patients had ischemic heart disease and hypertension, one participant (4%) had cardiac disease, one participant (4%) had cognitive dysfunction, and one person (4%) had hypothyroidism. Only two participants (8%) among the healthy group had asthma. Among CRC patients, cancer was found in 13 cases (52%) in the colon, 9 cases (36%) in the rectum, and 3 cases (12%) in the sigmoid. Thirteen CRC participants (52%) were in cancer stage III, and 12 patients (48%) were in cancer stage IV. Two cases (8%) of colon cancer and two cases (8%) of rectal cancer both progressed to metastasis.

### 3.2. Comparing Diet Quality: Exploring the Differences between CRC Patients and Healthy Controls

Among the 50 participants, only 15 and 10 participants of CRC and healthy controls were evaluated in terms of diet quality and fat intake. The remaining participants’ data concerning diet quality indicated a median score of 10.0 (9.00–11.0). Median diet quality scores of healthy participants and patients with CRC were similar. No statistical difference was also observed between healthy participants and patients with CRC in terms of the intake of fruits, vegetables, and fat (total fat, saturated fat, monounsaturated fat, polyunsaturated fat, fat from plant food sources, and fat from animal food sources). Detailed data concerning the diet quality and dietary intake of healthy participants and patients with CRC are presented in Table 2.

Multiple logistic regression analysis was performed to explore whether diet quality and fat intake can predict health status (CRC vs. healthy), adjusting for age, sex, and BMI. The results indicated that diet quality and dietary intake of fruits, vegetables, and fat (total fat, saturated fat, monounsaturated fat, polyunsaturated fat, fat from plant food sources, and fat from animal sources) were not linked to health status. The results of the multiple logistic regression analysis of CRC vs. healthy controls and diet quality and dietary intake are presented in Table 3.

### 3.3. Microbiome Study of Fecal Samples from CRC Patients and Healthy Controls

Based on 16S rDNA (V3-V4), amplicon sequencing analysis was carried out to study the microbial compositions of stool from CRC patients and the healthy controls. A total of 4,247,586 high-quality paired reads were produced from 50 sequenced samples, averaging 84,951.72 sequences per sample (minimum: 60,178; maximum: 102,003; median: 86,883.5). (Table S2). The distribution of these sequences was represented by 6052 OTUs (Table S3).

#### α and β Diversities of CRC vs. the Healthy Controls

**Figure 1:**
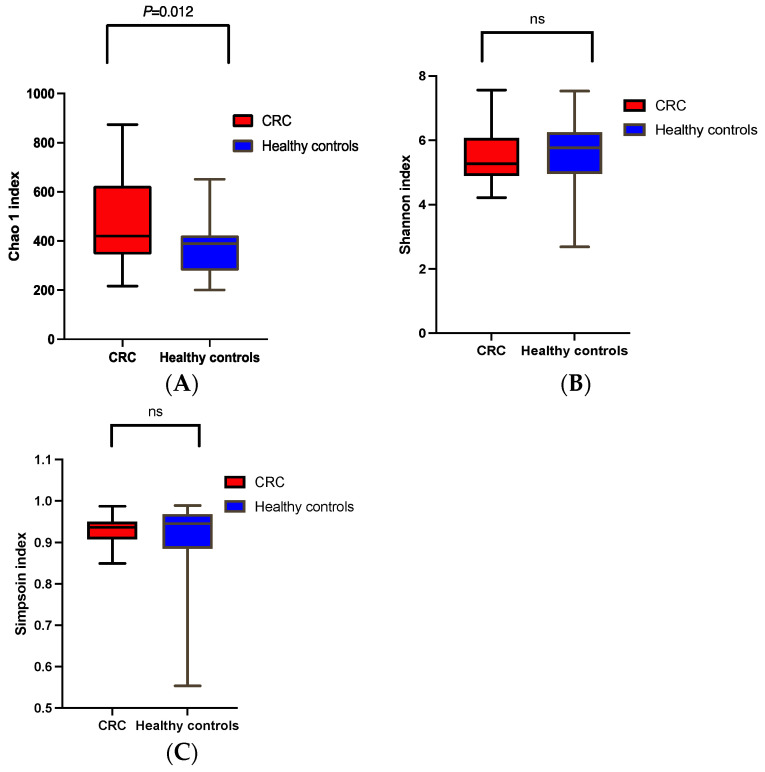
α and β diversity comparison between microbiomes collected from CRC patients and the healthy controls. Analysis was performed using sequencing data for the 16S rDNA V3 and V4. (**A**) Chao1, (**B**) Shannon, and (**C**) Simpson indices indicate α diversity. Whiskers in the boxplots represent the range of the minimum and maximum α diversity values within a population. CRC, colorectal cancer. Ns stands for non significant.

α and β diversities were analyzed using distinct indices. According to the Chao1 index, there was a significant difference between the α diversity of the CRC patients and the healthy controls (H: Chao 1 = 375.8 and CRC: Chao 1 = 484.7, *t*-test, *p* = 0.012), as shown in Figure 1A. However, a more equilibrated diversity such as Shannon and Simpson indices presented no significant difference between CRC patients and the healthy controls (H: Shannon = 5.578 and CRC: Shannon = 5.462, *t* test, *p* = 0.661; H: Simpson = 0.913 and CRC: Simpson = 0.929, and *p* = 0.390), as shown in Figure 1B,C.

**Figure 2:**
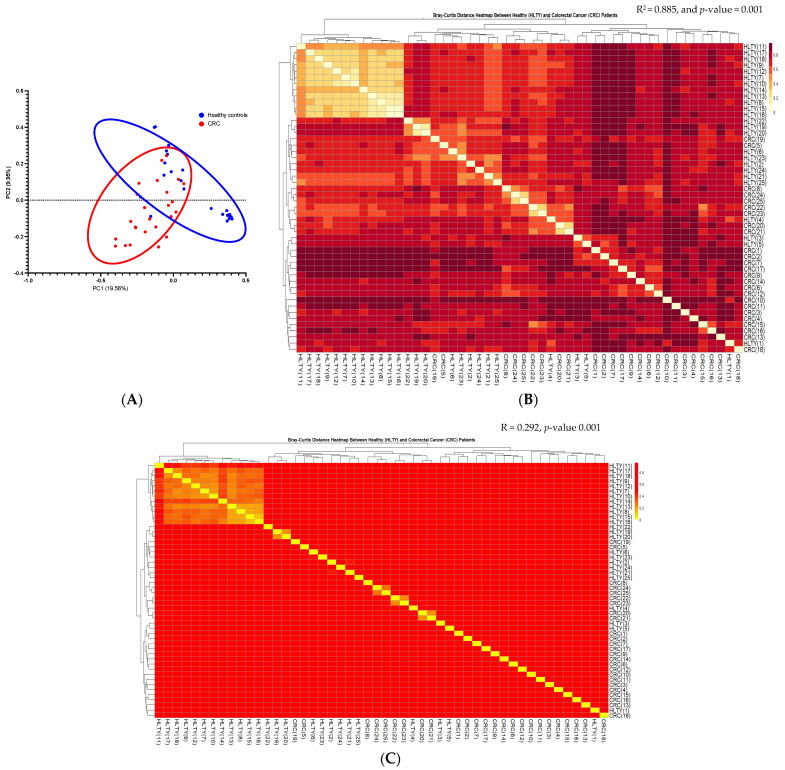
β diversity comparison between microbiomes collected from CRC patients and the healthy controls. (**A**) Principal coordinate analysis (PCoA) plot based on Bray–Curtis represented β diversity measured in unannotated OTUs composition. Red circles represent CRC patients, and blue circles represent the healthy control individual. (**B**) Heat map of adonis analysis based on Bray–Curtis represented β diversity measured in unannotated OTUs composition. CRC and healthy control samples were plotted in the *x*-axis and *y*-axis in the map. (**C**) Heat map of anoism analysis based on Bray–Curtis represented β diversity measured in unannotated OTUs composition. CRC and healthy control samples were plotted in the *x*-axis and *y*-axis on the map. CRC, colorectal cancer. Principal component 1 (PC1) and principal component 2 (PC2) were used.

The β diversity was determined using principal coordinate analysis (PCoA) based on Bray–Curtis analysis measured in the unannotated OTUs’ composition. As shown in Figure 2A, a clear clustering differentiated CRC and the healthy control samples. PERMANOVA analysis via the Bray–Curtis distance matrix indicated that the stool microbiome composition of CRC vs. the healthy controls was indeed different (F = 6.211, *p*-value 0.001) (Figure 2B). Anosim analysis showed that the stool microbiome composition between CRC and the healthy control groups was significantly larger than the variation within CRC or the healthy control’s microbiome (R = 0.292, *p*-value 0.001) (Figure 2C). Similarly, multi-response permutation procedure (MRPP) analysis indicated that the variation between CRC and the healthy controls was larger than the variation within groups (A = 0.0609, *p*-value 0.001). ADONIS or permutational MANOVA revealed that 88.5% of the variation in the distances was explained based on the grouping (CRC vs. the healthy controls) being tested (F = 6.21119).

### 3.4. Microbial Profile of CRC and Healthy Controls

The number of OTUs shared between CRC and the healthy participants was 603, whereas the number of OTUs that were unique to CRC and healthy participants were 2468 and 2952, respectively (Figure S1), indicating that the gut microbial composition of CRC shared few species with the healthy participants. The taxonomic assignment of the OTUs predicted for all the samples revealed the composition of their bacterial population at the phylum, family, and genus levels. Back to the phylum level, both CRC and the healthy controls’ fecal samples had a representation of *Firmicutes*, *Proteobacteria*, *Actinobacteriota*, *Bacteroidota*, *Verrucomicrobiota*, *Euryarchaeota*, *Fusobacteriota*, *Desulfobacterota*, *Patescibacteria*, and *Campilobacterota* (Figure S2A). At the family level, the following families were represented in both CRC and the healthy participants: *Lachnospiraceae*, *Peptostreptococcaceae*, *Streptococcaceae*, Enterobacteriaceae, *Leuconostocaceae*, *Lactobacillaceae*, *Bifidobacteriaceae*, *Enterococcaceae*, and *Comamonadaceae*. However, only *Porphyromonadaceae* was represented in CRC patients (Figure S2B). The bacterial genera that were abundant among CRC and healthy participants were *Romboutsia*, *Streptococcus*, *Weissella*, *Escherichia-Shigella*, *Lactobacillus*, *Bifidobacterium*, *Porphyromonas*, *Blautia*, *Enterococcus*, *Comamonas*, *Agathobacter*, *Holdemanella*, *Catenibacterium*, *Anaerostipes*, *Bacteroides*, *Collinsella*, *Eubacterium hallii group*, *Dorea*, *Pediococcus*, *Faecalibacterium*, *Parvimonas*, *Sarcina*, *Klebsiella*, *Subdoligranulum*, *Ruminococcus torques group*, *Roseburia*, *Intestinibacter*, *Erysipelotrichaceae UCG-003*, and *Turicibacter.* The two genera *Catenisphaera* and *Porphyromonas* are only present among CRC patients, albeit with low relative abundance (less than 0.01) (Figure S2C).

#### 3.4.1. The Most Abundant Bacteria in CRC vs. the Healthy Controls

**Figure 3:**
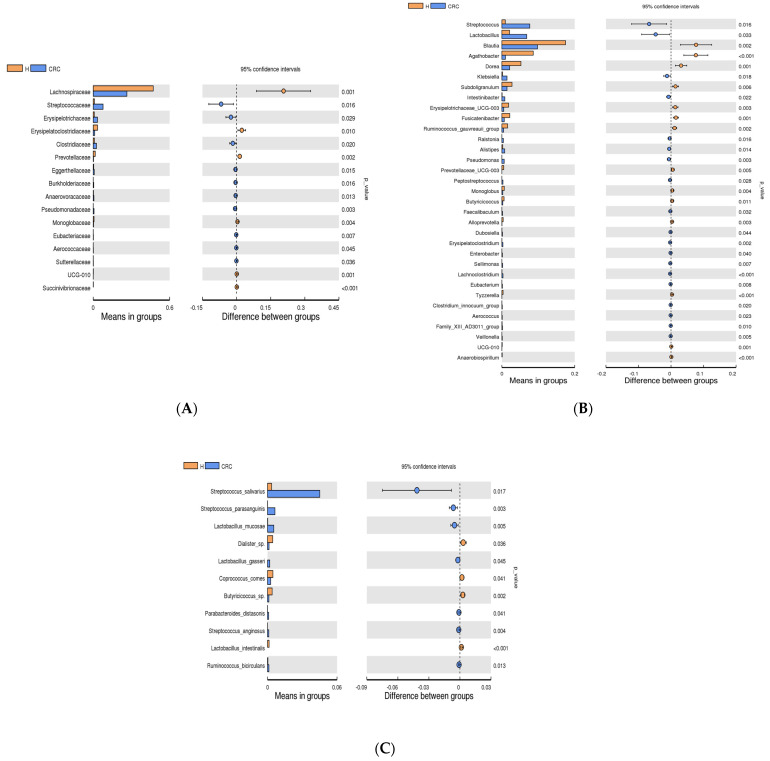
Differences between the identified family, genus, and species mean relative abundances per sample group comparison (CRC vs. the healthy controls). Bar graph (**A**–**C**) at the family, genus, and species levels. The left panel of the bar graphs (**A**–**C**) displays the abundances of families, genera, or species with significant variations between the CRC and the healthy controls. Each bar indicates the average mean abundance in each group of the sample, showing the differences between groups that are statistically significant. The confidential interval of between-group changes is shown in the right panel. Each circle’s outermost segment represents the lower limit of the 95% confidential interval, while the outermost segment represents the higher limit. The difference in the mean value is shown by the circle’s center. The circle’s color corresponds to the group whose mean value is higher. The *p*-value of the between-group variation significant *t*-test is the value on the right-hand side of the graph.

At the phylum level, the most abundant phyla (*Firmicutes*, *Proteobacteria*, *Actinobacteriota*, *Bacteroidota*, and *Verrucomicrobiota*) were not significantly different between CRC and the healthy participants (*t*-test, *p* > 0.05) (Table S4). Microbial relative abundance changes at the family, genus, and species level were addressed using bar graphs (Figure 3) (Tables S6 and S7). In the comparison at the family level, the top six individual families found in the control group were *Lachnospiraceae*, *Erysipelatoclostridaceae*, *Prevotellaceae*, *Monoglobaceae*, *Ruminococcaceae UCG-010*, and *Succiniribrionaceae* (*p*-value < 0.05) (Figure 3A) whereas in CRC, the significantly dominant families were *Streptococcaceae*, *Eryspelotrichaerthellaceae*, *Burkholderiaceae*, *Anaerovoraceae*, *Pseudomndaceae*, *Eubacteriaceae*, and *Aerococcaceae* (*p*-value < 0.05) (Figure 3A). The adjusted *p*-value by false discovery rate, with a Q-value < 0.05, also showed significant abundances of these families for CRC and the healthy participants (Table S5).

Figure 3B demonstrates that the microbial compositions changed at the genus level in CRC patients compared to the healthy controls. A significant difference was observed in 33 bacterial genera between the CRC group and the healthy control group. According to Figure 3B, the mean relative abundances of *Blautia*, *Agathobacter*, *Dorea*, *Subdoligranulum*, *Erypelotrichaceae* UCG-003, *Fusicatenibacter*, *Prevotellaceae*, *Monoglobus*, *Alloprevotella*, *Tyzzerella*, Oscillospirales-UCG-010, and *Anaerobiospirillum* were found to be significantly increased (upregulated) in the healthy control group compared to CRC (*p* < 0.05) (Q < 0.05, as shown in Table S6). On the other hand, *Streptococcus*, *Lactobacillus*, *Klebsiella*, *Intestinibacter*, *Ralstonia*, *Alistipes*, *Pseudomonas*, *Peptostreptococcus*, *Faecalibaculum*, *Dubosiella*, *Erysipelatoclostridium*, *Enterobacter*, *Sellimonas*, *Lachnoclostridium*, *Eubacterium*, *Clostridium innocuum*, *Aerococcus*, Family_XIII_AD3001 Group, and *Veillonella* were found to be enriched (upregulated) in CRC compared to the healthy controls (*p* < 0.05) (Figure 3B) (Q < 0.05, as shown in Table S6).

The most significantly abundant bacterial species in the healthy controls were *Dialister* species, *Coprococcus comes*, *Butyricicoccus* species, and *Lactobacillus intestinalis* (*p* < 0.05) (Figure 3C) (Q < 0.05, as shown in Table S7). However, among CRC patients, the most abundant species were *S. salivarius*, *S. parasanguins*, *S. anginosus*, *L. mucosae*, *L. gasseri*, and *Parabacteroides distasonis* (*p* < 0.05) (Figure 3C) (Q < 0.05, as shown in Table S7).

#### 3.4.2. Characterization of the Microbiomes of CRC Patients and Healthy Participants via LEfSe Analysis and LDA Based on OTUs

**Figure 4:**
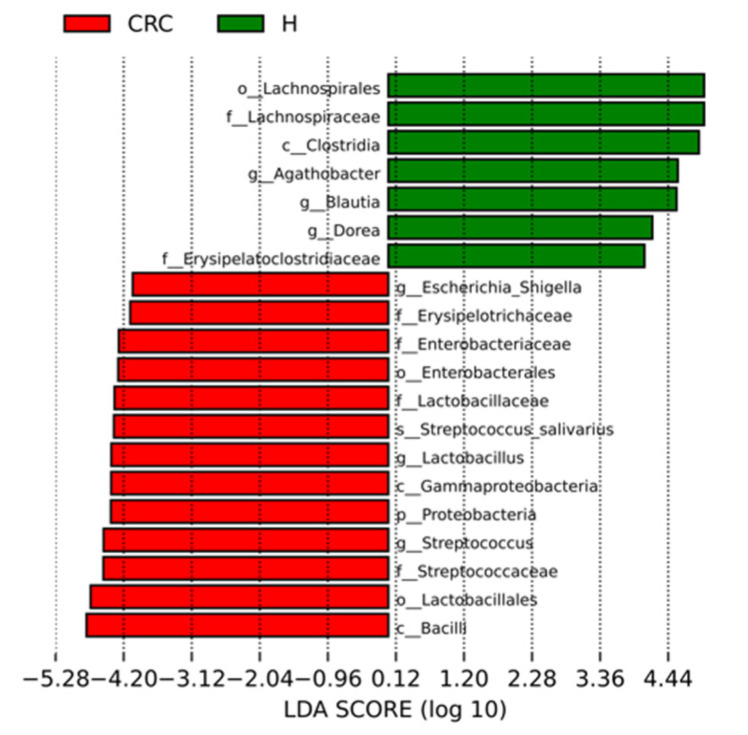
Microbiome markers characterization in CRC vs. healthy control using LEfSe analysis and LDA. Histograms of the LDA scores (log 10) computed for features with differential abundances in CRC vs. the healthy controls. H stands for healthy controls, and CRC stands for colorectal cancer.

The estimated phylotypes of patients with CRC and healthy microbiota were then compared using LEfSe analysis. A histogram of the LDA scores was created for features that displayed different abundances in healthy participants compared to CRC patients. According to the LDA scores, CRC patients had significantly higher relative abundances of *Esherichia-Shigella*, *Ersipelotichaceae*, *Enterobacteriaceae*, *Enterobacterales*, *Streptococcus salivarus*, *Gammaproteobacteria*, *Proteobacteria*, *Streptococcus*, *Streptococcaceae*, and *Lactobacillales* (LDA score [log 10] > 4), whereas the healthy subjects’ microbiome was characterized by a predominance of *Lachnopirales*, *Lachnospiraceae*, *Clostridia*, *Agathobacter*, *Blautia*, and *Dorea* (LDA score [log10] > 4) (Figure 4).

#### 3.4.3. Diet Quality and Fat Intake Association with Most Abundant Bacteria Genera among CRC

Simple linear regression analysis was performed to investigate the association between diet quality and the most abundant bacteria in CRC patients. The analysis suggested a link between *Alistipes* and diet quality among participants with CRC (*p* = 0.029; R-square = 0.47), whereas no other bacteria were linked to diet quality (Table 4). No association was observed between total fat intake and the most abundant bacteria in CRC patients (Table 5).

### 3.5. Functional Enrichment Analysis and Pathway Abundance Differences in CRC Compared to Healthy Controls

**Figure 5:**
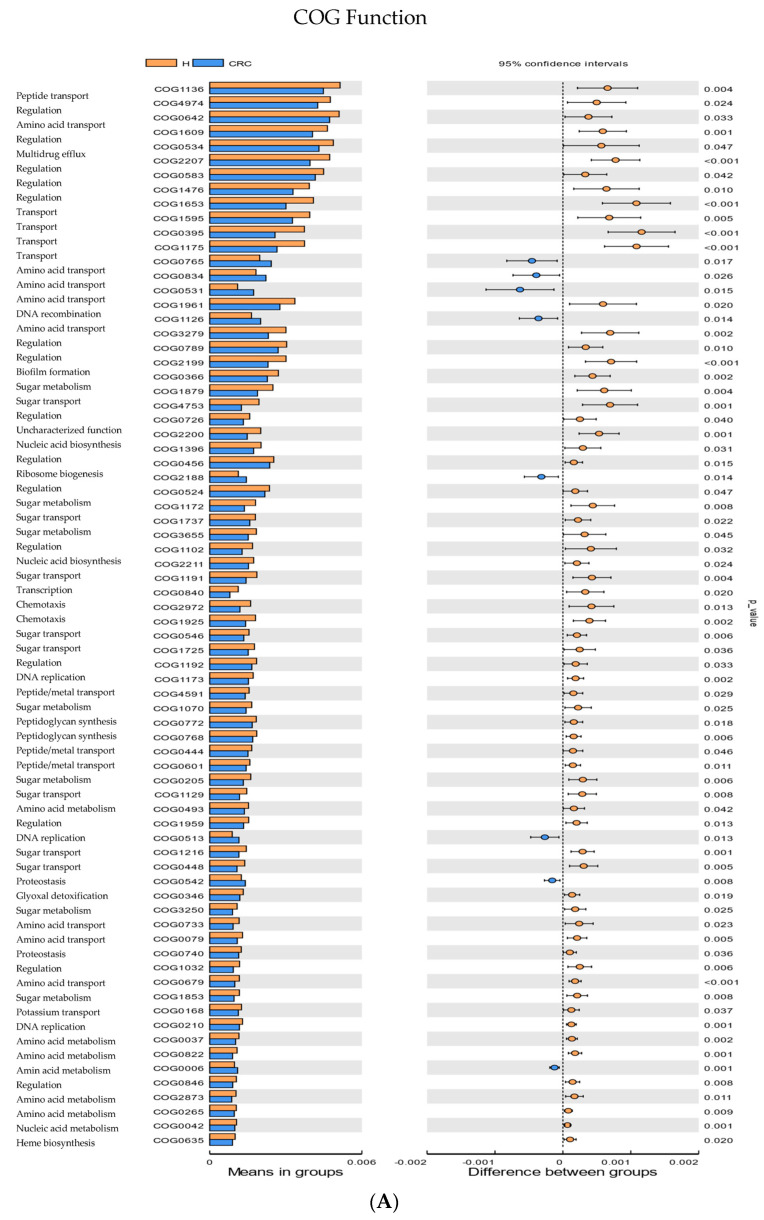

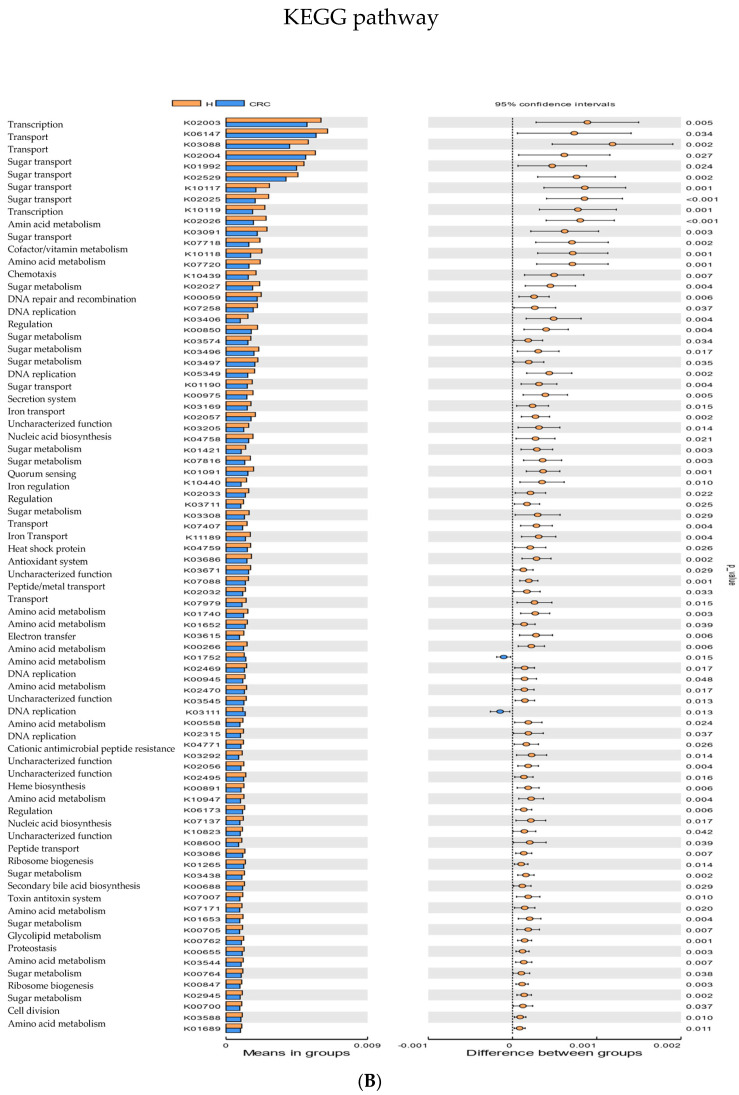
The function prediction of CRC vs. the healthy controls. (**A**) The bar graph represents the differences in the cluster of ortholog genes’ (COG) function. (**B**) The bar graph shows the abundance differences of the KEGG pathway. *t*-test analysis was used to compare the two groups (CRC vs. healthy control), and a *p*-value < 0.05 was marked as significant.

The functional prediction of the 16S rRNA was implemented using the PICRUST2 program, and the results of the cluster of ortholog genes’ (COG) functional annotation and the abundance difference of the Kyoto Encyclopedia of Genes and Genomes (KEGG) pathway revealed 2602 and 3566 functions and pathways that were shared between CRC and healthy controls, respectively (Figure S3A,B). However, 36 and 63 COG and KEGG predicated functions and pathways were unique for CRC patients, and 38 and 93 were specific for healthy participants (Figure S3A,B). The analysis revealed that these functions were distinctly enriched in CRC compared to the healthy controls in ABC-type amino acid transport system, permease component (COG0765, *p* = 0.017, Q = 0.027), ABC-type amino acid transport/signal transduction system, periplasmic component/domain (COG0834, *p* = 0.026, Q = 0.033), Serine transporter YbeC, amino acid: (COG0531, *p* = 0.014, 0.025), ABC-type polar amino acid transport system, ATPase component (COG1126, *p* = 0.014, Q = 0.024), DNA-binding transcriptional regulator, GntR family (COG1126, *p* = 0.014, Q = 0.024), Superfamily II DNA and RNA helicase (COG0513, *p* = 0.013,Q = 0.024), ATP-dependent Clp protease, ATP-binding subunit ClpA (COG0542, *p* = 0.008, Q = 0.018), and Xaa-Pro aminopeptidase (COG0006, *p* = 0.001, Q = 0.007) (Figure 5A, Figure S4A) All the *p*-values were adjusted via FDR, and the Q-values were significant, i.e., Q < 0.05, as shown in Table S8. We found two main pathways that were enriched in CRC vs. the healthy controls, according to the KEGG database. They were connected to glycine metabolism, serine and threonine metabolism, cysteine and methionine metabolism (K01752, *p* = 0.015, Q = 0.023), and DNA replication and mismatch repair (K03111, *p* = 0.013, Q = 0.022) (Figure 5B, Figure S4B). All the *p*-values were adjusted via FDR, and the Q-values were significant, i.e., Q < 0.05, as shown in Table S9.

## 4. Discussion

More than a trillion microbiota cells are found in the colorectum, producing a significant variety of small molecules (i.e., metabolites), which regulate a number of critical pathways involved in energy homeostasis, nutrient uptake, and immune balance [35]. Additionally, growing evidence suggests that the CRC’s microbiome and associated metabolome play a role in carcinogenesis [36]. Microbiota dysbiosis can break down and/or modify carbohydrates, proteins, and lipids to create oncometabolites and tumor suppressor metabolites, key players in CRC’s occurrence and development [16].

In the present study, we first documented a significantly lower diversity of the microbiota in advanced stages of cancer (III and IV) compared to the healthy controls based on the different α and β diversity indices (Figure 1A and Figure 2A–C). Similarly, Gao’s study [37] revealed a significant difference between patients with advanced precancerous lesions and those with advanced stages of cancer and healthy controls. However, Feng [38] and Yacgida’s studies [39] reported no significant differences between patients with advanced precancerous lesions and healthy controls. The discrepancy may be partially explained by the difference in ethnic background and sample size.

Diet is one of the most significant modifiable factors of gut microbiome. The diversity in dietary practices among various populations can cause microbiota variations [40]. Our study found no significant associations between diet quality, fat intake, and health status (CRC vs. healthy). Both groups exhibited poor diet quality. This can be explained by the limited variability in the diet consumed by Saudis and the small sample size. Previous research investigating the diet of Saudis indicated high consumption of fat and limited consumption of whole grains, fruits, vegetables, fish, and milk. In contrast, the consumption of lower-quality foods, such as processed meats and sugary drinks, was high. Previous research investigating the diet of Saudis indicated high consumption of fat and limited consumption of whole grains, fruits, vegetables, fish, and milk. In contrast, the consumption of lower-quality foods, such as processed meats and sugary drinks, was high [41]. A study conducted in Saudi Arabia comparing the gut microbiome between Westernized urban Saudis and non-industrialized rural Saudis found that the Westernized urban Saudis’ diets had significantly lower richness and biodiversity than non-industrialized rural Saudis [42].

The distribution of the gut microbiota at the genus level exhibited variations between CRC and healthy participants. The most predominant signature microbiota in the healthy participants includes (i) bacteria associated with maintaining a healthy gut and (ii) bacteria linked to poor diet or metabolic diseases. The bacteria identified in the healthy participants, namely *Blautia*, *Agathobacter*, *Dorea*, *Subdoligranulum*, *Prevotellaceae*, *Monoglobus*, and Alloprevotela (Figure 3C), have been associated with the healthy gut microbiome, as reported in previous studies [43,44,45,46,47]. *Blautia* species is one of the common intestinal microbes linked to exhibiting potential probiotic effects, as reviewed by Liu [43]. However, few studies’ data associated this genus with metabolic illnesses and inflammatory diseases [48]. It is still unclear how the prevalence of *Blautia* contributes to these diseases; however, the connection between *Blautia* and diseases may depend on specific species or strains. *Agathobacter* can produce butyrate from acetate and maintain healthy gut ecology and homeostasis [44]. *Dorea*, *Alloprevotela*, *Blautia*, and *Subdoligranulum* contribute significantly to maintaining a healthy gut by producing different propositions of SCFAs, such as butyrate, propionate, and acetate, as reported previously [44,49]. A recent study from New Zealand pointed to *Monoglobus*, specifically *M. pectinilyticus*, as the first identified bacteria with pectin-degrading enzymatic systems in the human colon [47]. Members of *Prevotellaceae* and *Prevotella* were positively associated with a high-fiber diet and protection against *Bacteroides*-induced glucose intolerance, contributing positively to glucose metabolism [46].

Among the most signature microbiota in our healthy participants, certain bacteria have been associated with poor diet or metabolic diseases, such as *Fusicatenibacter*, *Tyzzerella*, *Oscillospirales-UCG-010*, and *Anaerobiospirillum* (Figure 3C). It has been shown that *Fusicatenibacter* is associated with poor eating habits [50]. In our study, we found that diet quality was poor for both the CRC and healthy groups. *Alloprevotela* was linked to metabolic disorders due to poor diet quality [11]. The relatively high abundance of *Tyzzerella* was connected to saturated fatty acid (SFAs) and trans fatty acid (TrFAs) intake [49]. This bacterium has been linked to cardiovascular diseases [49,51]. *Oscillospiraceae UCG010* was enriched among the healthy participants, and *Oscillospiraceae NK4A214* has been identified previously as a prognostic marker for obesity [52]. However, it was reported in a recent study that *Oscillospiraceae UCG010*, among other bacteria, such as *Bacteroides*, *Clostridium*, *Parabacteroides*, *Christensenella*, *Oscillospira*, and *Ruminococcaceae UBA1819*, were the most dominant microbes among non-obese microbiota [53]. *Anaerobiospirillum* is an emerging human intestinal microbiota [54], as it has been known to colonize cats and dogs [55]. However, this bacterium has been isolated recently from patient’s feces with diarrhea [55]. In our study, *Erysipelotrichaceae UCG-003* was enriched among the healthy participants, and this bacterium has been found to be the most abundant microbe among healthy ageing participants [56]. The healthy group’s mean age was 47 ± 11.7 in our study. All in all, the gut micorbiome profile of the healthy participants in Saudi has been associated with two types of microbes, namely microbes associated with maintaining healthy gut and microbes linked to poor diet or metabolic diseases.

Next, the microbiome profiling of our CRC patients identified enrichment in *S. salivarius*, *S. parasanguins*, *S. anginosus*, *L. mucosae*, *L. gasseri*, *Peptostreptococcus*, *Eubacterium*, *Aerococcus*, Family XIII_AD3001 Group, *Erysipelatoclostridium*, *Escherichia-Shigella*, *Klebsiella*, *Enterobacter*, *Alistipes*, *Ralstonia*, and *Pseudomonas*. The collective impact of these bacteria can have an essential role in the metabolite profile, contributing to the progression of CRC. The intestinal microbial community influences the metabolism processes for a wide range of compounds. For example, gut bacteria’s contribution to the metabolism of bile acids (BAs) was found to be critical for maintaining human health [57]. The main biotransformation pathways of bile acid are (i) bile salt hydrolase (BSH), hydrolyzing conjugated bile acids to free bile acids and glycine or taurine, (ii) 7-dehydroxylation of cholic acid (CA) and chenodeoxycholic acid (CDCA) to produce deoxycholic acid (DCA) and lithocholic (LCA), and (iii) bile acid 7-dehydroxylation of ursodeoxycholic acid (UDCA) to form LCA [57]. Previous studies have proposed that the imbalance between primary and secondary BAs is strongly linked to CRC, and the increase in the secondary BA pool has a critical role in CRC development [17].

Various secondary BA-associated bacteria were identified among the CRC fecal samples in this study. The two families, namely *Erysipelotrichaceae* and *Lactobacillacae*, that belong to the *Firmicutes* phylum were found to be significantly enriched in our CRC patients (Figure 4). The *Erysipelotrichaceae* family has been found to play a role in secondary BA transformation [41,42,43]. The significant role of *Erysipelotrichaceae* bacteria has been documented in gastrointestinal illnesses associated with inflammation [58]. For instance, Chen’s study [58] reported that the abundance of this family was higher in the lumen of colorectal cancer patients compared to healthy controls. In our study, two *Lactobacillus* species, namely *L. mucosae* and *L. gasseri*, significantly increased among CRC fecal samples. A recent finding showed that *Lactobacillus* species have evolved to deconjugate bile acids in the gut [59]. This study claimed that 28% of the investigated *Lactobacilli* encode BSH proteins, a key enzyme involved in secondary BA biotransformation [59]. A previous study [60] was conducted to analyze the change in microbial composition in CRC subjects with both fecal microbiota and gut microbe-derived extracellular vesicles (EVs). This study linked the elevated levels of *Lactobacillus* to the early stage of CRC [60]. Furthermore, *Peptostreptocoocus* and *Eubacterium* were significantly increased in our CRC patients (Figure 3B), and they were found to express an enzyme that contributes to secondary BA transformation [61]. These bacteria encode the enzyme 7 beta-hydroxysteroid dehydrogenase, which transforms UDCA to LCA [61]. Gao’s [37] findings documented that patients diagnosed with CRC were significantly enriched in *Peptostreptococcus*, along with other bacterial species such as *Fusobacterium*, *Parvimonas*, *Enterococcus*, and *Escherichia/Shigella*. *E. rectale* promotes colitis, contributing to the development of CRC [62]. A recent study identified Family XIII AD3001 Group as one of the bacteria that are strongly associated with BA metabolism [63], and this group was identified as one of the significantly upregulated bacteria found in our CRC patients. The complete characterization of the secondary BA metabolites, which could be generated by these bacteria, and their role in the occurrence and development of CRC is warranted to understand their role in CRC development and allow early CRC diagnosis, which can benefit human health.

Another metabolite that has been associated with CRC and found to be produced by gut microbiota is lactate. Lactate levels in the feces of healthy people are generally low [64]. Beneficial species of *Clostridia* are the main lactate consumers in the gut [64]. Among other microbes, *Clostridia* can create butyrate, which is the primary fuel for colonocytes. In the absence of butyrate, colonocytes move from fatty acid metabolism to glucose metabolism, which results in an increase in lactate in the gut lumen [64]. Lactate encourages tumor growth and development by providing energy to tumor cells and evading immune defenses [18]. *C. innocuum* was one of the bacteria enriched in our CRC (Figure 3B). It can produce lactate when fatty acid metabolism is slowing down [64,65]. Another bacterial group is *Lactobacilli*, well characterized as producing lactic acid and metabolizing lactic acid into lactate [66]. Moreover, the family *Enterobacteriaceae* includes many genera of bacteria, such as *Salmonella*, *Shigella*, *Escherichia coli*, *Proteus*, and *Klebsiella* [67], that can produce lactate [68]. *Escherichia-Shigella* and *Klebsiella* were found to be increased in our CRC patients (Figure 3B and Figure 4). These bacteria are typically commensal in humans’ guts, despite being opportunistic pathogens. According to recent studies [69,70,71], CRC patients had higher levels of *Enterobacteriaceae* than healthy individuals. *Escherichia-Shigella* species frequently inhabit the colon and could potentially foster tumor growth, thereby establishing these genera as potential risk factors for CRC [72]. *Klebsiella* species was another *Enterobacteriaceae* bacterium that produced lactate and showed elevated levels among our CRC sample (Figure 3B). Previous studies [73,74] have connected *Klebsiella* species, particularly *K. pneumoniae*, to causing pyogenic liver abscess (PLA) in CRC. According to the comparison between the two groups, patients with *K. pneumoniae*-PLA had a significantly greater incidence rate of CRC [73,74].

Important human opportunistic pathogen *Streptococcus* species (Figure 3B), particularly *S. salivarius*, *S. parasanguins*, and *S. anginosus* (Figure 3C), were significantly enriched among our CRC. These bacteria can produce lactate [68]. Additionally, *Streptococcus* species can express pro-carcinogenic traits associated with pro-inflammatory activities, including leucocytic recruitment, the potential for specific adhesion to tumor tissues, and the selective colonization of tumor cells. These characteristics establish an optimal microenvironment for tumor tissues downstream [75,76,77]. In other studies [75,78], *S. bovis*, later named *gallolyticus*, has been linked to CRC. *S. bovis*/*gallolyticus* has been discovered to be responsible for the slow-moving carcinogenesis of colon mucosal tissues. This suggests the potential use of *S. bovis*/*gallolyticus* for detecting colorectal carcinomas through the detection of *S. bovis*/*gallolyticus* DNA or their particular IgG antibodies. This will provide a significant advantage in screening high-risk populations for CRC. Another bacteria identified among our CRC fecal samples was *Aerococcus*, which is also a member of lactate-producing bacteria group [79]. Only one paper linked *Aerococcus* to CRC [80], which highlights the need to understand the role of this bacteria in CRC pathophysiology.

A genotoxic metabolite that can be produced by intestinal commensal–pathogenic bacteria is colibactin. Colibactin is a secondary metabolite expressed by polyketide *synthase* (*pks*) island [66,81]. Three bacteria, such as *Escherichia*, *Klebsiella*, and *Enterobacter*, were identified in our CRC as being able to can encode *pks* and express colibactin, as documented previously. In preclinical models and cell lines, *E. coli* carrying this biosynthetic gene cluster induces DNA damage and cancer [66,81]. A variety of bacteriocins have been found in an in vitro investigation that can encourage the development of tumor cells. Among *Enterobacteriaceae pks*-positive bacteria carrying the *pks* genes was a risk factor in the development of CRC [82,83,84]. The bacterial toxin colibactin encoded in *pks* genes was also found to be conserved in particular *K. pneumoniae* strains [85]. Hence, *pks* positive *K. pneumoniae* can be employed specifically in a commercial biomarker panel to discover CRC prevalence in high-risk populations, such as PLA patients. Additionally, *Enterobacter* carries *pks* genes and expresses colibactin [86], which was significantly profiled among our CRC fecal samples. However, previous studies [66,81,85,86], have documented the production of colibactin in particular strains of *E. coli*, *Klebsiella*, and *Enterobacter*. Our study did not confirm the production of colibactin by these identified bacteria. Further characterization is warranted to verify colibactin production.

According to our findings, *Alistipe* was upregulated among CRC patients and had a positive correlation with poor diet quality (Table 3). This bacterium belongs to the phylum *Bacteroidetes*, which is predominantly linked to chronic intestinal inflammation [87]. There is strong evidence indicating the role of *Alistipe* in CRC, chronic diseases, and mental health, as reviewed in [87]. This bacterium has been isolated from different clinical samples, suggesting their role as opportunistic pathogens [88]. Recently, sulfanolipid is one of the metabolites that have been found to be synthesized by *Alistipe* species, residing in mammalian gut [89]. A study has claimed that sulfanolipid can activate renal cell line carcinoma [90]. The role of sulfanolipid in CRC is unknown, highlighting the need to characterize its role in CRC development. Our study did not investigate whether *Alistipe* produces sulfanolipid; however, the role of sulfanolipid-producing bacteria and sulfanolipid in CRC pathophysiology is unknown, which underlines the need for further research.

Cancer patients are more susceptible to invasive infections by opportunistic pathogens and Gram-negative bacteria because of the ulcerative lesions on their mucosal surfaces and the immunological suppression brought on via chemotherapy [91,92]. *Pseudomonas species* and *Ralstonia species* had elevated levels in our CRC patients (Figure 3B). *P. aeruginosa* intestinal carriage rises from 3% in healthy individuals to 20% in hospitalized patients [93]. According to the epidemiological findings [94,95], *P. aeruginosa* intestinal colonization is common among cancer patients who are hospitalized. All CRC cases examined in this study pertained to advanced stages of the disease. This circumstance elucidates the prevalence of *Pseudomonas* and *Ralstonia* among CRC patients.

Two bacterial genera, namely *Sellimonas* and *Lachnoclostridium*, were upregulated among our CRC fecal samples. Studies have documented the abundance of these bacteria among CRC and the potential use of these bacteria for early CRC diagnosis [96,97]. However, more studies are needed to further characterize their causal role in CRC pathophysiology. Among our CRC fecal samples, we identified four bacteria, namely *Faecalibaculum*, *Dubosiella*, *Parabacteroides distasonis*, and *Veillonella*, that were significantly enriched among CRC (Figure 3B), although they were already known for their anti-carcinogenic effect on tumor progression and decreased abundance in microbial dysbiosis [98,99,100]. Studies have reported the expansion of *Faecalibaculum* populations due to specific diets, specifically a high-calcium phosphate diet [101], and drug interactions, such as treatment with Metformin, an antidiabetic drug [102]. The cause of the high relative abundance of the *Faecalibaculum* population among our CRC group is unknown. Numerous studies documented the anti-tumorigenic activity of *Dubosiella* [103]. It has been reported that *D. newyorkenesis* is enriched with high nitrite-containing sausage [104]. Some studies have linked the high nitrite diet to an increased risk of CRC; however, a few studies found contradicting results, as reported by Crowe et al. [105]. *P. distasonis* has been profiled in two studies as a passenger microbiota in the CRC’s mucosal tissue samples in Indian [106] and Thia’s [106] CRC patients. *Veillonella* has been associated with gut microbial dysbiosis in CRC patients who had an appendectomy [107]. These bacteria are also found to be enriched when lactic acid bacteria are commonly abundant in the gut [108,109]. In our CRC fecal sample, we have an over-representation of *Lactobacilli*; however, further investigation is needed to understand their role in CRC.

In this study, we further investigated the functional enrichment analysis and pathway abundance differences in our CRC compared to the healthy participants. The majority of functions and pathways are reduced in individuals with CRC (Figure 5A,B). With a few exceptions, the CRC group has more of the functions related to amino acid transport, signaling and metabolism, membrane biogenesis, DNA replication and mismatch repair, and protease activity. Numerous studies have demonstrated the interaction between the host and gut microbiome [110,111]. In fact, cancer cells use the majority of essential metabolic pathways, such as glucose, glutamine, amino acids, serine/glycine, and lipid metabolism, to maintain their high rates of cell division [112]. Bacterial proteases activity has been linked to a number of diseases, such as inflammatory bowel disease (IBD) and celiac disease (CeD). For instance, *P. aeruginosa*’s elastase-like activity can cause the formation of peptides after gluten metabolism with enhanced immunogenicity in CeD patients [113]. In a mouse model of spontaneous colitis, the metalloprotease GelE, generated by *Enterococcus faecalis*, breaks down E-cadherin, causing a loss of barrier function, which has been discovered previously in inflammation cases [114]. The microbiota can transform compounds from the diet into DNA-damaging agents, and some bacteria can also produce such toxins when there is dysbiosis. Dysbiosis microbiota can break down carbohydrates, proteins, and lipids to create oncometabolites and tumor suppressor metabolites, which have negative effects on the immune system, epigenetic regulation, and genotoxicity [16]. Pathogenic bacteria that produce a toxin that damages DNA that may be present in disturbed microbiota and dysbiosis situations [115]. A recent review performed by [116] indicated the role of DNA damage pathways generated by a diet-modified microbiome, suggesting a possible avenue to create new methods and treatments to lower the risk of CRC development.

This study has certain limitations, despite its new findings. We, therefore, outlined the study’s strengths and constraints. The strengths of this research were as follows: (1) this study marks a pioneering effort in delineating the distinctive gut microbiome associated with CRC within the Saudi population; (2) only advanced CRC cases were included; (3) the results of microbial profiling and LDA demonstrated that bacterial community differed among the two groups; and (4) we discovered that there were different dominant bacteria among CRC patients and healthy participants, as well as the enrichment predicated functions among CRC. The following points are the study’s limitations: (1) the sample size was not excessive; (2) the generalizability of our findings may be limited due to the small sample size; (3) our reliance on self-reported dietary intake and the small sample size included in the diet data introduce potential recall bias and affect the statistical inferences; (4) diet quality and dietary intake were only performed on random samples of the CRC cases; and (5) further characterization of the microbial-derived metabolites is required among CRC cases. In future research, the metagenomics and metabolomics techniques should be used to ascertain a study’s predicted function. Larger sample sizes and longitudinal studies are needed for more robust insights. Implementing more rigorous dietary assessment methods would enhance the accuracy and reliability of results. Additionally, it is crucial to evaluate the role of metabolites in the development of CRC.

## 5. Conclusions

In conclusion, this study’s findings indicate differences in the microbial composition and functional potential of the gut microbiota between CRC patients and healthy controls. We discovered a significant increase in *S. salivarius*, *S. parasanguins*, *S. anginosus*, *L. mucosae*, *L. gasseri*, *Peptostreptococcus*, *Eubacterium*, *Aerococcus*, Family XIII_AD3001 Group, *Erysipelatoclostridium*, *Escherichia-Shigella*, *Klebsiella*, *Enterobacter*, *Alistipes*, *Ralstonia*, and *Pseudomonas*. Only *Alistipes* was significantly linked to poor diet quality in CRC. Further research is needed to better understand the role of the gut microbiome and its metabolites in CRC development and progression, including larger cohorts, longitudinal studies, and investigations into potential causative mechanisms.

## Figures and Tables

**Table 1:** Characteristics of CRC patients and healthy controls included in this study.

Characteristics	Healthy Controls N = 25 (50%)	CRC Patients N = 25 (50%)
Sex		
Male	21 (84.0)	15 (60.0)
Female	4 (16.0)	10 (40.0)
Age (yrs)		
Mean ± SD	47.4 ± 11.7	54.3 ± 14.2
Weight status		
Underweight	0 (0.00)	2 (8.00)
Healthy weight	16 (64.0)	12 (48.0)
Overweight	2 (8.00)	9 (36.0)
Obesity	7 (28.0)	2 (9.00)
Cancer stage		
I		0 (0.00)
II		0 (0.00)
III		13 (52.0)
IV		12 (48.0)
Metastasis		4 (16.0)
Cancer location		
Sigmoid		3 (12.0)
Colon		13 (52.0)
Rectum		9 (36.0)

Data presented in the table are frequency, percentage %, and mean ± standard deviation (SD).

**Table 2:** Diet quality and dietary intake of CRC patients and healthy controls included in this study.

	Total (n = 25)	Healthy Controls (n = 15)	CRC Patients (n = 10)	*p*-Value
Diet quality score	9.48 ± 1.11 10.0 (9.00–11.0)	9.67 ± 1.11 10.0 (9.00–11.00)	10.1 ± 1.10 10.0 (9.00–11.00)	0.495
Fruits, servings/day	1.60 ± 0.50 2.00 (1.00–2.00)	1.53 ± 0.52 2.00 (1.00–2.00)	1.70 ± 0.48 2.00 (1.00–2.00)	0.495
Vegetables, servings/day	1.44 ± 0.58 1.00 (1.00–2.00)	1.33 ± 0.49 1.00 (1.00–2.00)	1.60 ± 0.70 1.50 (1.00–2.00)	0.428
Total fat, g/day	83.00 ± 41.40 76.00 (52.50–104)	83.30 ± 36.10 77.0 (54.00–100)	82.60 ± 50.60 69.0 (39.00–140)	0.531
Saturated fat, g/day	29.50 ± 15.40 25.00 (16.50–39.5)	29.10 ± 14.00 25.0 0(18.00–30.0)	30.00 ± 18.00 23.50 (13.50–51.00)	0.683
Monounsaturated fat, g/day	30.80 ± 17.30 26.00 (16.50–42.50)	30.40 ± 13.30 28.00 (22.00–38.00)	31.50 ± 22.80 25.50 (11.00–51.80)	0.683
Polyunsaturated fat, g/day	14.30 ± 8.500 12.00 (7.50–20.00)	14.10 ± 6.55 13.00 (9.00–20.00)	14.60 ± 11.20 11.00 (5.00–24.50)	0.567
Fat from plant food sources, g/day	39.40 ± 27.0 30.00 (15.00–57.00)	37.9 ± 20.3 36.0 (22.00–57.00)	41.6 ± 35.9 23.0 (13.00–66.50)	0.723
Fat from animal food sources, g/day	43.60 ± 22.30 43.00 (27.50–50.00)	45.2 ± 20.20 43.0 (30.00–49.00)	41.20 ± 26.10 35.5 (21.00–68.00)	0.397

Data presented in the table are mean ± standard deviation and median (interquartile range). α = 0.05. The Mann–Whitney U test was used to compare groups.

**Table 3:** Multiple logistic regression analysis of diet quality and dietary intake among CRC patients and healthy controls included in this study.

	Odds Ratio (OR)	95% Confidence Interval	* p- * Value
Diet quality score	0.40	0.06 to 2.70	0.348
Fruits, servings/day	0.11	0.003 to 4.34	0.236
Vegetables, servings/day	0.46	0.02 to 9.18	0.608
Total fat, g/day	0.99	0.94 to 1.03	0.558
Saturated fat, g/day	1.01	0.92 to 1.12	0.798
Monounsaturated fat, g/day	0.94	0.82 to 1.07	0.340
Polyunsaturated fat, g/day	0.90	0.70 to 1.14	0.370
Fat from plant food sources, g/day	0.97	0.89 to 1.05	0.422
Fat from animal food sources, g/day	1.00	0.92 to 1.07	0.912

All models were adjusted for age, sex, and body mass index. α = 0.05.

**Table 4:** Simple linear regression model of diet quality and the most abundant bacteria at the genus level in CRC patients in comparison to healthy controls included in this study.

Bacteria	Healthy Controls			CRC Patients		
	B (SE)	*p*-Value	R-Square	B (SE)	*p*-Value	R-Square
*Streptococcus*	0.00 (0.00)	0.859	0.00	−0.05 (0.04)	0.203	0.19
*Lactobacillus*	0.00 (0.01)	0.757	0.01	0.02 (0.02)	0.468	0.07
*Klebsiella*	*	*	*	*	*	*
*Intestinibacter*	*	*	*	*	*	*
*Ralstonia*	*	*	*	*	*	*
*Alistipes*	0.00 (0.00)	0.159	0.15	0.01 (0.00)	0.029 *	0.47
*Pseudomonas*	*	*	*	1.567 × 10^−5^ (0.00)	0.692	0.02
*Peptostreptococcus*	*	*	*	0.00 (0.00)	0.853	0.01
*Faecalibaculum*	*	*	*	*	*	*
*Dubosiella*	*	*	*	*	*	*
*Erysipelatoclostridium*	*	*	*	*	*	*
*Enterobacter*	*	*	*	*	*	*
*Sellimonas*	*	*	*	*	*	*
*Lachnoclostridium*	*	*	*	2.618 × 10^−5^ (0.00)	0.063	0.37
*Eubacterium*	5.560 × 10^−5^ (0.00)	0.225	0.11	−1.137 × 10^−5^ (0.00)	0.320	0.12
*Clostridium*	0.00 (0.00)	0.061	0.25	8.491 × 10^−5^ (0.00)	0.839	0.01
*Aerococcus*	*	*	*	*	*	*
Family XIII AD3011 group	*	*	*	*	*	*
*Veillonella*	*	*	*	0.00 (0.00)	0.820	0.01

SLR, Simple linear regression model, α = 0.05. * SLR data are not presentable due to the small figures.

**Table 5:** Simple linear regression analysis of fat intake and the most abundant bacteria at the genus level in CRC patients in comparison to healthy controls included in this study.

Bacteria	Healthy Controls			CRC Patients		
	B (SE)	*p*-Value	R-Square	B (SE)	*p*-Value	R-Square
*Streptococcus*	4.981 × 10^−5^ (0.00)	0.327	0.07	0.00 (0.00)	0.303	0.13
*Lactobacillus*	0.00 (0.00)	0.457	0.04	0.00 (0.00)	0.467	0.07
*Klebsiella*	*	*	*	*	*	*
*Intestinibacter*	*	*	*	*	*	*
*Ralstonia*	*	*	*	*	*	*
*Alistipes*	−5.251 × 10^−6^	0.818	0.00	−5.948 × 10^−5^ (0.00)	0.456	0.07
*Pseudomonas*	*	*	*	−4.883 × 10^−7^ (0.00)	0.569	0.04
*Peptostreptococcus*	*	*	*	−4.403 × 10^−5^ (0.00)	0.218	0.18
*Faecalibaculum*	*	*	*	*	*	*
*Dubosiella*	*	*	*	*	*	*
*Erysipelatoclostridium*	*	*	*	*	*	*
*Enterobacter*	*	*	*	*	*	*
*Sellimonas*	*	*	*	*	*	*
*Lachnoclostridium*	*	*	*	−6.919 × 10^−8^ (0.00)	0.840	0.01
*Eubacterium*	−3.448 × 10^−7^ (0.00)	0.813	0.00	−2.281 × 10^−7^ (0.00)	0.361	0.11
*Clostridium*	2.200 × 10^−8^ (0.00)	0.992	0.00	1.296 × 10^−5^ (0.00)	0.124	0.27
*Aerococcus*	*	*	*	*	*	*
Family XIII AD3011 group	*	*	*	*	*	*
*Veillonella*	*	*	*	1.724 × 10^−5^ (0.00)	0.090	0.32

SLR, Simple linear regression model, α = 0.05. * SLR data are not presentable due to the small figures.

## Data Availability

The data presented in this study are openly available in the Sequence Read Archive (SRA) of the National Centre for Biotechnology Information (NCBI), under the submission reference number SUB13865299 (https://submit.ncbi.nlm.nih.gov/subs/sra/SUB13865299, accessed on 15 June 2023).

## Supplementary Materials

The following supporting information can be downloaded at: https://www.mdpi.com/article/10.3390/cancers15205019/s1.
