# Supplementary material for: Identification of Gut Microbiota Profile Associated with Colorectal Cancer in Saudi Population
Source: Cancers (Basel). 2023 Oct 17;15(20):5019. doi: 10.3390/cancers15205019 (PMC10605194; doi:10.3390/cancers15205019)
Supplement: Supplementary file 1 [file cancers-15-05019-s001.zip › Figures S1-S4.pdf]

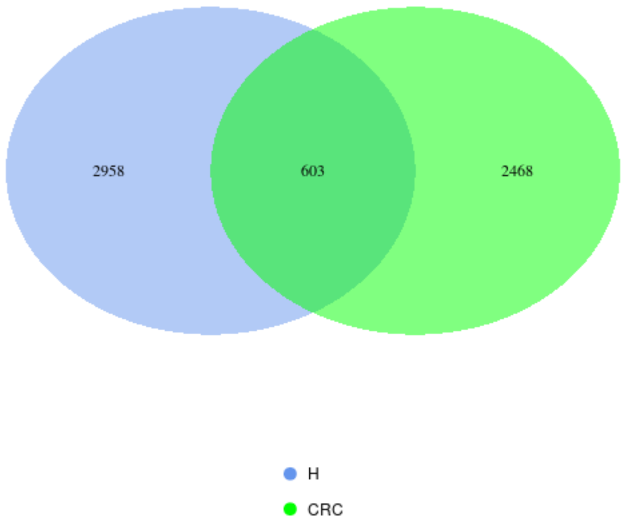

Figure S1: Venn diagram of OUTs for CRC and healthy participants (Green: CRC and blue: healthy participants)

A

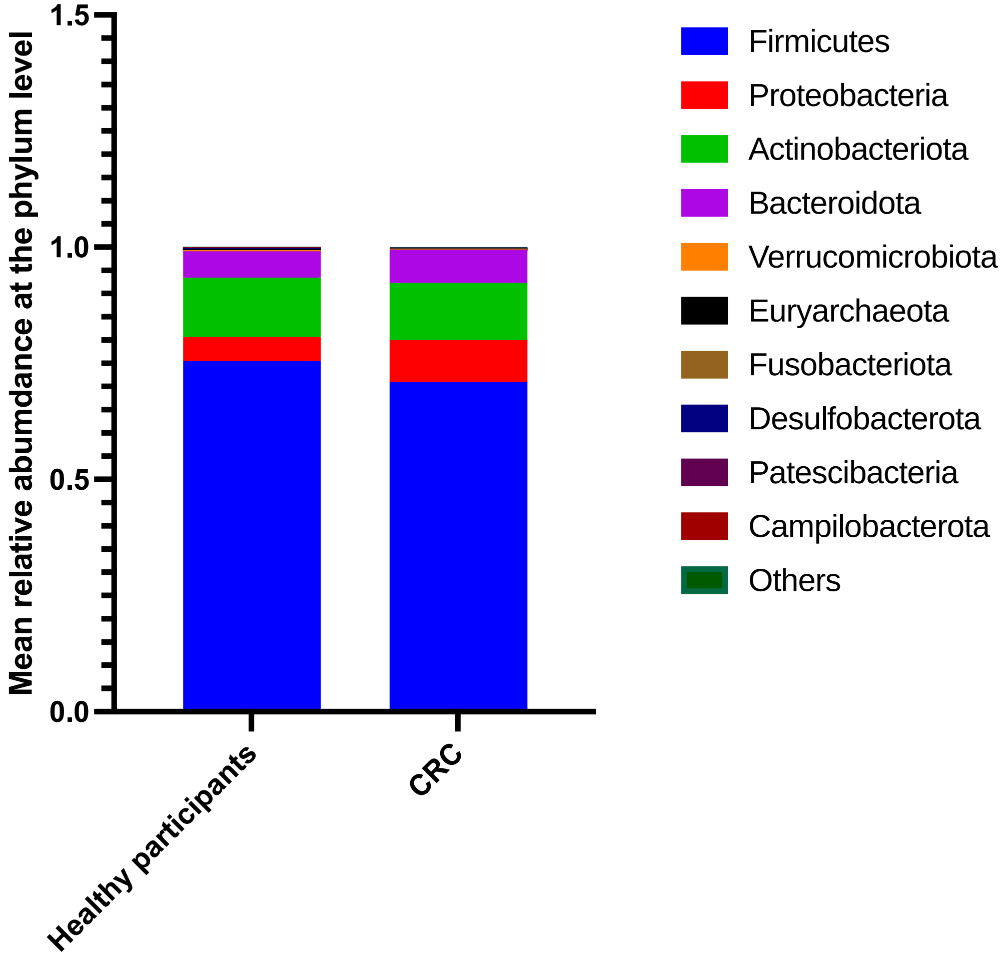

Mean relative abumdance at the phylum level
1.5
1.0
0.5
0.0
Healthy participants
CRC
Firmicutes
Proteobacteria
Actinobacteriota
Bacteroidota
Verrucomicrobiota
Euryarchaeota
Fusobacteriota
Desulfobacterota
Patescibacteria
Campilobacterota
Others

B

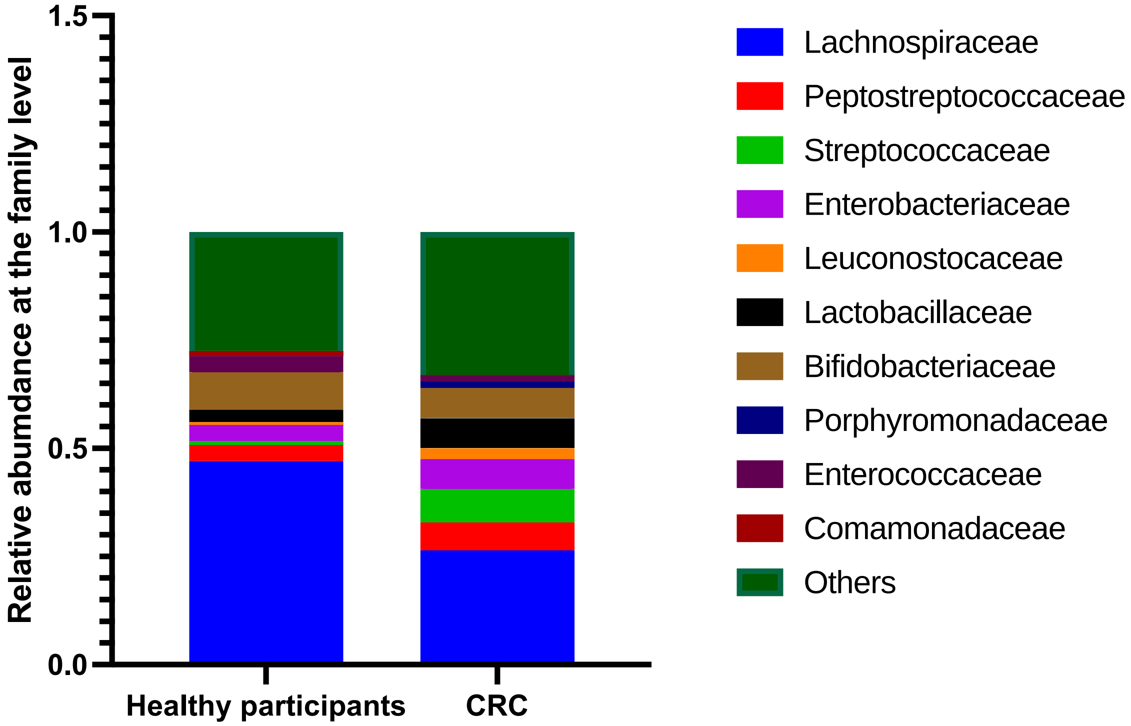

Relative abumdance at the family level
1.5
1.0
0.5
0.0
Healthy participants
CRC
Lachnospiraceae
Peptostreptococcaceae
Streptococcaceae
Enterobacteriaceae
Leuconostocaceae
Lactobacillaceae
Bifidobacteriaceae
Porphyromonadaceae
Enterococcaceae
Comamonadaceae
Others

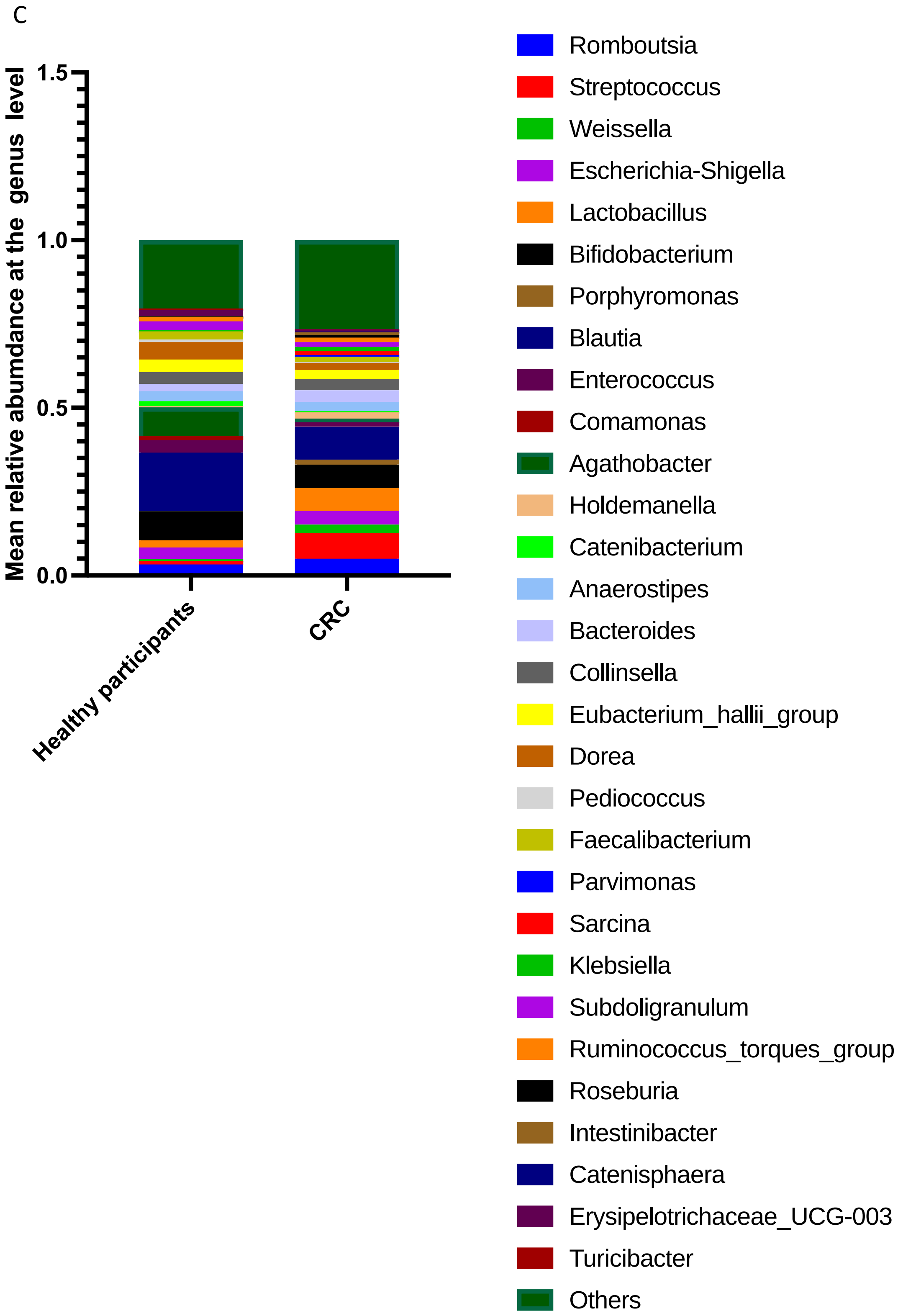

Figure S2: The taxonomic composition distribution of gut microbiota. (A) Relative abundance of bacterial phyla in gut microbiota in CRC and the healthy participants. (B) Relative abundance of bacterial family in gut microbiota in CRC and the healthy participants. (C) Relative abundance of bacterial genera in gut microbiota in CRC and the healthy participants.

A

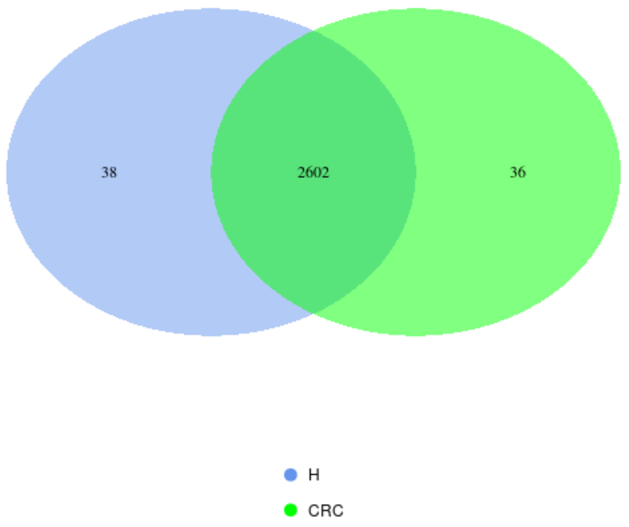

B

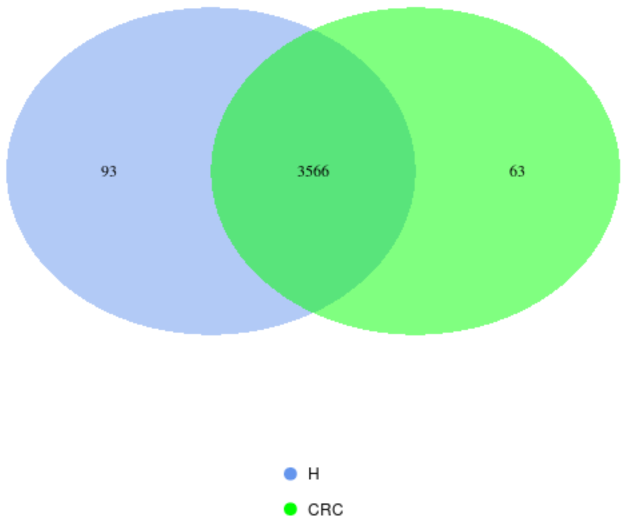

Figure S3: Venn diagram of (A) COG and (B) KEGG predicated functions and pathways for CRC and healthy participants (Green: CRC and blue: healthy participants)

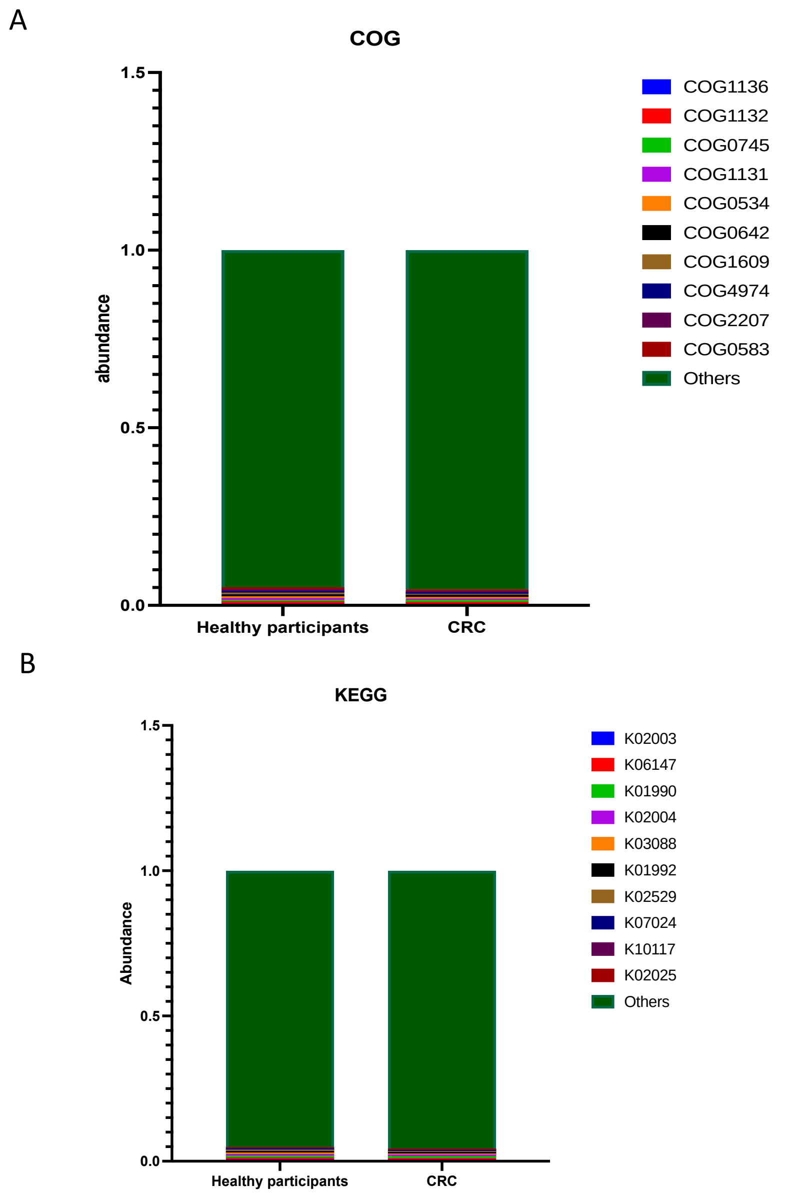

Figure S4: The predicated functions and pathways among CRC patients and the healthy participants. (A) Abundance of the predicated COG functions in 16S rRNA genes in the healthy participants and CRC. (B) Abundance of the predicated KEGG pathways in 16S rRNA genes in the healthy participants and CRC.
